# A Rare Case Report of Fanconi Anemia-Associated Nuclease 1 Mutation Causing Nephrotic Syndrome

**DOI:** 10.7759/cureus.64041

**Published:** 2024-07-07

**Authors:** Raja Sekar, Ilakyaa Rajakumar, Gerry G Mathew, Tanuj Lamech, Varadharajan Jayaprakash

**Affiliations:** 1 Nephrology, SRM Medical College Hospital and Research Centre, Chengalpattu, IND

**Keywords:** fanconi anemia-associated nuclease 1 mutation, african ancestry, chronic kidney disease, focal segmental sclerosis, nephrotic syndrome

## Abstract

A 25-year-old African male patient presented with a history of frothy urination for one month. He had a significant family history of early onset chronic kidney disease (CKD) in his older brother. On evaluation, he was found to have deranged renal function and nephrotic-range proteinuria of 6152 mg/day. Urine examination revealed proteinuria and glycosuria. Viral serology and autoimmune screening results were negative. Ultrasonography revealed contracted kidneys that were not amenable to biopsy. Genetic analysis revealed a Fanconi anemia-associated nuclease 1 (FAN 1) mutation in exon 4 (c.1399G>A) and exon 12 (c.2786A>C). The patient was managed conservatively with a maximum dose of angiotensin receptor blockers with a reduction in proteinuria on follow-up. This case report highlights the rare manifestation of FAN 1 mutation and its variable effects on the kidney.

## Introduction

Fanconi anemia-associated nuclease 1 (FAN 1) mutation is a rare genetic mutation associated with karyomegalic interstitial nephritis (KIN) and various neurodevelopmental disorders [1,2]. FAN 1 mutation causes KIN, autism spectrum disorders, epilepsy, cancer, and various other neurocognitive disorders [1,2]. The usual renal presentation includes a mild degree of proteinuria, hematuria, or chronic kidney disease (CKD) [3]. Here we describe a rare renal presentation of FAN 1 mutation and the possible pathogenetic mechanisms behind this clinical manifestation.

## Case presentation

**Figure 1:**
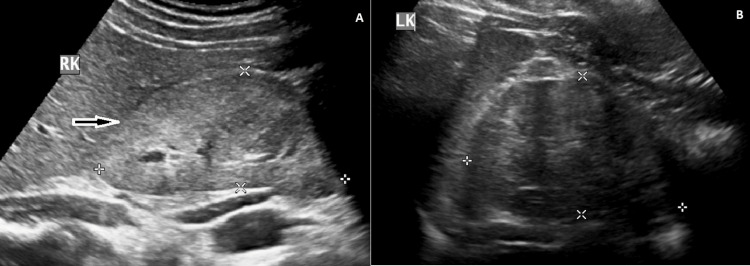
A: ultrasound showing right kidney measuring 8.8 x 4.1 cm with increased echogenicity and lost CMD (white-bordered black arrow (magnification: 600 DPI)) and B: ultrasound showing contracted left kidney measuring 8.5 x 5.3 cm with lost CMD (white-bordered black arrow (magnification: 600 DPI)) CMD: corticomedullary distinction; DPI: dots per inch

A 25-year-old male from Nigeria of African ancestry was admitted with a history of frothy urination and pedal edema for one month. He had a strong family history of end-stage renal disease (ESRD) in his family, with the elder brother having CKD stage 5D at the age of 30 years who underwent renal transplant and succumbed to postoperative complications. On evaluation, the patient was found to have accelerated blood pressure of 210/120 mmHg, grade 3 hypertensive retinopathy, and concentric left ventricular hypertrophy on echocardiography. He had no evidence of renal bruit and had normal peripheral pulses. On laboratory evaluation, his creatinine was 1.8 mg/dL (0.74-1.35 mg/dL), urea was 48 mg/dL (5-20 mg/dL), random blood sugar was 108 mg/dL (<125 mg/dL), normal electrolytes, normal liver enzymes, serum protein was 6.7 g/dL (6-8.3 g/dL), and serum albumin was 3.4 g/dL (3.5-5 g/dL). His lipid profile revealed a serum cholesterol level of 202 mg/dL (<200 mg/dL) and a serum triglyceride level of 168 mg/dL (<150 mg/dL). Urine examination revealed 3+ albumin and 2+ glucose with 1-2 RBCs/high power field. A 24-hour urine protein level was 6152 mg/day. Ultrasonography revealed a right kidney measuring 8.8 cm (Figure 1A) and a left kidney measuring 8.5 cm (Figure 1B) with raised echogenicity and lost corticomedullary distinction (CMD).

Renal Doppler imaging revealed no evidence of renal artery stenosis. Serological examination revealed negative results for hepatitis B, hepatitis C, HIV, and antinuclear antibodies. He had normal complement levels, and a renal biopsy was not attempted because of the loss of CMD and contracted kidneys. Due to a strong family history of ESRD, genetic analysis using next-generation sequencing revealed a compound heterozygous FAN 1 mutation in exon 4 (c.1399G>A) (p.Glu467 Lys) and exon 12 (c.2786A>C)(p.Tyr929Ser). The exon 4 mutation is responsible for glutamic acid to lysine substitution and the exon 12 mutation is responsible for tyrosine to serine substitution. Genetic analysis of the distant family could not be performed because of logistic reasons. The patient was started on the maximum dose of the angiotensin receptor blocker, telmisartan (80 mg/day), and atorvastatin (20 mg/day) for lipid control. At three month follow-up, his 24-hour urine protein level reduced to 1950 mg/day, with a stable lipid profile and a creatinine level of 2 mg/dL.

## Discussion

FAN 1 encodes a deoxyribonucleic acid (DNA) repair nuclease, and mutations in this gene are implicated in KIN, neurocognitive disorders such as autism, epilepsy, schizophrenia, repeat expansion diseases, Rett syndrome, and cancer [1]. FAN 1 protein has nuclease activity, and mutations in this gene would result in increased susceptibility of renal tissue to genotoxic stress, environmental insults, and impaired DNA repair, thereby leading to CKD progression by initiating the pathognomonic process of renal fibrosis [2]. KIN is a very rare hereditary chronic interstitial nephritis characterized by karyomegalic cells lining the proximal and distal convoluted tubules that lead to ESRD below the age of 50 years [3]. FAN 1 mutation affects the kidney because the gene and the gene products are abundantly expressed in the renal tissue indicating the protective function of FAN 1 nuclease protein in maintaining the structural and functional integrity of renal tissues [2,3].

KIN is characterized by slowly progressive CKD with a mild degree of proteinuria and hematuria [1-3]. We strongly suspect that our patient had KIN due to the strong family history of early adult-onset ESRD in the elder brother in light of the FAN 1 mutation on next-generation sequencing genetic analysis and the presence of glucosuria, which is evident in 75% of the patients afflicted by this disorder [3]. The presence of glycosuria in our patient is evidence of proximal tubular dysfunction induced by abnormal karyomegalic cells lining the proximal tubules [4]. The uniqueness of our case was the presence of nephrotic syndrome in our patient. Radha et al. raised the possibility of sporadic focal segmental sclerosis in a KIN patient with renal biopsy to explain the presence of nephrotic syndrome [5]. However, the common embryological lineage of tubular epithelial cells and podocytes raises the possibility of an unexplained genotoxic insult instigated by the FAN 1 mutation, which may be responsible for the nephrotic syndrome and faster progression to ESRD, as evidenced by the strong family history of our index patient [6]. 

The unique feature of this case is a good response to conventional anti-proteinuria management with angiotensin receptor blockers without the requirement for immunosuppression, contrary to previously published case reports [5,6]. This clinical outcome emphasizes the variable effects and outcomes of FAN 1 mutation in different racial diasporas, which warrants further research. The drawback of our case report is the lack of renal biopsy, which would have shed light on the underlying histopathology and pathogenesis of the FAN 1 mutation in our patient. This is probably the first reported case of FAN 1 mutation causing nephrotic syndrome and CKD in a patient of African ancestry, raising the possibility of other novel mutations besides the apolipoprotein L1 gene mutation (APOL 1) in CKD pathogenesis among African diaspora [7]. Nevertheless, this case report is an eye-opener to investigate the complex interplay between different protective genes and their role in the progression of CKD.

## Conclusions

FAN 1 mutation presenting as nephrotic syndrome with CKD is a rare clinical entity and this differential diagnosis has to be considered while evaluating patients of African lineage besides the conventional APOL1 mutation. Focal segmental glomerulosclerosis progressing to CKD should be considered as an important renal differential of FAN 1 mutation, which needs early evaluation and management.
